# Sagittal plane gait characteristics in hip osteoarthritis patients with mild to moderate symptoms compared to healthy controls: a cross-sectional study

**DOI:** 10.1186/1471-2474-13-258

**Published:** 2012-12-20

**Authors:** Ingrid Eitzen, Linda Fernandes, Lars Nordsletten, May Arna Risberg

**Affiliations:** 1 The Norwegian Research Center for Active Rehabilitation (NAR)/Orthopaedic Department, Oslo University Hospital, Oslo, Norway; 2 Orthopaedic Department, Oslo University Hospital, Oslo, Norway; 3 The Norwegian Research Center for Active Rehabilitation/The Norwegian School of Sports Sciences/Orthopaedic Department, Oslo University Hospital, Oslo, Norway

**Keywords:** Osteoarthritis, Hip, Biomechanics, Gait analysis

## Abstract

**Background:**

Existent biomechanical studies on hip osteoarthritic gait have primarily focused on the end stage of disease. Consequently, there is no clear consensus on which specific gait parameters are of most relevance for hip osteoarthritis patients with mild to moderate symptoms. The purpose of this study was to explore sagittal plane gait characteristics during the stance phase of gait in hip osteoarthritis patients not eligible for hip replacement surgery. First, compared to healthy controls, and second, when categorized into two subgroups of radiographic severity defined from a minimal joint space of ≤/>2 mm.

**Methods:**

Sagittal plane kinematics and kinetics of the hip, knee and ankle joint were calculated for total joint excursion throughout the stance phase, as well as from the specific events initial contact, midstance, peak hip extension and toe-off following 3D gait analysis. In addition, the Western Ontario and McMaster Universities Osteoarthritis Index, passive hip range of motion, and isokinetic muscle strength of hip and knee flexion and extension were included as secondary outcomes. Data were checked for normality and differences evaluated with the independent Student’s t-test, Welch’s t-test and the independent Mann–Whitney U-test. A binary logistic regression model was used in order to control for velocity in key variables.

**Results:**

Fourty-eight hip osteoarthritis patients and 22 controls were included in the final material. The patients walked significantly slower than the controls (*p*=0.002), revealed significantly reduced joint excursions of the hip (*p*<0.001) and knee (*p*=0.011), and a reduced hip flexion moment at midstance and peak hip extension (*p*<0.001). Differences were primarily manifested during the latter 50% of stance, and were persistent when controlling for velocity. Subgroup analyses of patients with minimal joint space ≤/>2 mm suggested that the observed deviations were more pronounced in patients with greater radiographic severity. The biomechanical differences were, however, not reflected in self-reported symptoms or function.

**Conclusions:**

Reduced gait velocity, reduced sagittal plane joint excursion, and a reduced hip flexion moment in the late stance phase of gait were found to be evident already in hip osteoarthritis patients with mild to moderate symptoms, not eligible for total hip replacement. Consequently, these variables should be considered as key features in studies regarding hip osteoarthritic gait at all stages of disease. Subgroup analyses of patients with different levels of radiographic OA further generated the hypothesis that the observed characteristics were more pronounced in patients with a minimal joint space ≤2 mm.

## Background

As the hip is crucial for locomotion [1,2], hip osteoarthritis (OA) is one of the leading causes for gait impairments in the elderly population [3-5]. Research on biomechanics during gait in hip OA has, however, up until now focused on the end-stage of disease [6], with the vast majority of existent studies describing gait characteristics before and after total hip replacement (THR) [7-17]. Overall, there is consistency in reporting that hip OA patients reveal some form of gait alterations. The most frequently reported deviations are reduced stride length and reduced cadence, reduced gait velocity, and reduced joint excursion; in particular reduced hip extension in the sagittal plane [18-24]. However, a recent meta-analysis by Ewen et al. [18] underlined that gait analyses on hip OA have not been conducted in a consistent manner. Including seven articles; a total of 46 different biomechanical variables were reported. This illustrates the lack of a clear consensus on which specific gait parameters should be regarded as being of most relevance for patients with hip OA [19,25,26]. Consequently, the role of gait biomechanics in hip OA initiation and progression is not fully understood. For patients with early stage hip OA, the number of studies is far less [27,28], emphasizing this challenge even more. However, as it has been argued that adequate exercise and muscular strengthening can contribute to reduce abnormal joint loading in hip OA, and, hence, impede disease progression [29], early identification of deviant gait characteristics is crucial in order to develop targeted rehabilitation interventions for patients with hip OA.

The main aim of the present study was to explore sagittal plane gait characteristics in subjects with hip OA with mild to moderate symptoms compared to healthy controls. We hypothesized that the hip OA patients would reveal reduced hip excursion during stance, and a reduced gait velocity compared to the healthy subjects. As a secondary analysis, we wanted to explore potential differences in gait between the hip OA patients when categorized into two subgroups based on their level of radiographic OA (ROA) severity. Pain is recognized as a cardinal symptom of hip OA [30-32], but self-reported symptoms are not always well associated with the degree of joint degeneration [1,33]. Thus, we wanted to investigate whether there were differences in biomechanical characteristics between patients with different levels of ROA. Jacobsen and colleagues [34] found a minimal joint space (MJS) of ≤2 mm to be the radiographic criterion most strongly associated to self-reported hip pain. Furthermore, ≤2 mm MJS is the established cut-off used by the orthopedic surgeons at our clinic when categorizing patients with hip OA from their level of radiographic progression. Therefore, we pre-defined MJS ≤2 mm and >2 mm as cut-offs for the subgroups, and hypothesized that the patients with more severe ROA would reveal reduced hip excursion during stance, and also reduced gait velocity compared to those with less severe ROA.

As the novelty of this study entailed an explorative approach, we included The Western Ontario and McMaster Universities Osteoarthritis Index (WOMAC) subscales for pain, function and stiffness, clinically assessed range of motion (ROM) of the hip joint, and isokinetic muscle strength of the extensor and flexor muscles of the hip and knee joints as secondary assessments to describe the level of symptoms and function of the subjects included in our material.

## Material and methods

### Subjects

**Figure 1:**
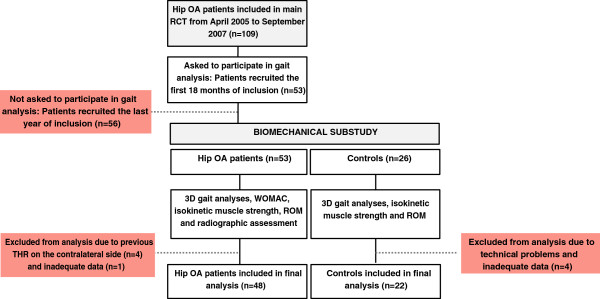
Flow-chart of the material included in the study.

This study was a biomechanical substudy from a larger randomized controlled trial (RCT) (http://www.clinicaltrials.gov; reference number NCT00319423) where 109 patients with hip OA with mild to moderate pain were included [35]. The aim of the overall RCT was to evaluate the efficacy of adding an exercise program to patient education, with WOMAC pain as primary outcome. The present material involved a convenience sample of the first 53 patients included in the RCT, who in addition to clinical and functional assessments also went through a data collection with biomechanical 3D motion analysis. Motion analyses were in addition conducted for 26 healthy controls. As all the patients were referred to baseline gait analyses before they were allocated to randomization, this substudy had a cross-sectional design. A flow-chart of the material in relation to the overall RCT is shown in Figure 1.

Patients were recruited from two hospitals, one sports medicine and rehabilitation center, and from family physician clinics. Patients between 40 and 80 years with uni- or bilateral hip pain for at least three months were eligible for inclusion. Subjects who agreed to participate were examined according to the Harris Hip Score (HHS) [36], and assessed by the same physical therapist (LF). As a HHS score <60 is used as a cut-off for THR at our institution [37], and >95 reflects normal function, a HHS score range from 60–95 was interpreted to reflect mild to moderate symptoms and set as an inclusion criterion. The main HHS-score of the included patients was 78.2 (SD 8.2). Subjects were further assessed with radiographs, and hip ROA was verified following Danielsson’s criteria [38]. For subjects with bilateral involvement, the most painful hip joint was defined as the target joint. Previous THR, recent trauma and/or injury involving the lower limb, rheumatoid arthritis, cancer, heart disease, osteoporosis, low back pain or knee pain led to exclusion from the study. The controls were recruited through acquaintances of the included patients, and of colleagues at our institution. To be included, controls had to be within the same age limits, have a HHS score ≥96 and withstand the same exclusion criteria as the hip OA patients. All participants signed an informed consent before inclusion. The study was approved by the Regional Medical Research Ethics Committee of Eastern Norway, and conducted in accordance with the Helsinki Declaration.

### Outcome measurements

#### Gait analysis

Gait analyses took place in the Motion Analysis Laboratory at The Norwegian School of Sports Sciences. Kinematic data was collected using a Qualisys pro-reflex motion analysis system (Qualisys AB, Gothenburg, Sweden) with eight cameras at a sampling frequency of 240 Hz, synchronized with three AMTI LG6 force plates (Advanced Mechanical Technology Inc, Watertown, MA, US) sampling kinetic data at a rate of 960 Hz. Reflective passive anatomical markers defining the joint centers were placed over the medial and lateral malleolus, medial and lateral femoral condyle, bilaterally over the greater trochanter, and bilaterally on the top iliac crest. Further, three rigidly attached reflective passive markers placed on thermoplastic shells were located at the sacrum, and bilaterally at the thigh and shank. Both feet were defined by two markers attached to the heel of the shoe and one marker at the 5^th^ metatarsal head. Subjects were instructed to look straight forward and walk at their self-selected speed along a 17 meter walkway, in which the force plates were embedded. Self-selected walking speed has been shown to be preferable in order to reduce the variability of gait [39]. Velocity was measured by photoelectric beams located 3.06 m apart, midway along the walkway. Data collection was continued until 12 acceptable hits for each limb on the force plates were captured. As 5–10 trials have been suggested to assure adequate reliability and prevail inherent variability in gait analyses in patients with hip OA [40], we selected the 6–8 trials of the 12 captured that were free from artifacts and within +/− 5% of the average velocity for analyses. Data were processed with Visual 3D software (C-motion Inc, Crabbs Branch Way Rockville MD), a movement analysis program which calculates the six degrees of freedom of a link rigid segment in an inertial reference system and uses that information to compute joint kinematics and kinetics. The following events were defined during the stance phase of gait: Initial contact (threshold 25 N), midstance (identified as the midpoint temporal observation of the stance phase when normalized from 0-100%), peak hip extension (peak hip extension angle) and toe-off (threshold 25 N).

Ten of the 48 patients that were included in the final analyses had bilateral involvement. There were no significant systematic differences between these patients and the patients with unilateral involvement on any of the outcome variables (*p*>0.05). Thus, only the limb with the target hip joint of the patients was included in the analyses. Further, there were no systematic differences between the right and the left limb of the controls (*p*>0.05). Therefore, the left limb was drawn to be included as control limb. Total sagittal plane joint excursion of the hip, knee and ankle joint was calculated throughout the stance phase of gait, as well as joint angles (°) and joint moments given as external moments in Newton-meters normalized to bodyweight in kilograms (Nm/BW) for the hip, knee and ankle joint at the four defined events.

#### Radiographic assessment

MJS of the target joint was measured on standardized postero-anterior digital pelvic radiographs (Syngo Imaging V36, Siemens AG, Erlangen, Germany) centered on the symphysis. Assessment of the MJS have shown to be a valid approach in diagnosing hip OA [2]. All radiographs were evaluated by one orthopedic surgeon (LN). The shortest distance between subchondral bone in the femoral head and in the acetabulum was identified visually, and the distance to the femoral surface measured in millimeters (mm).

#### Functional and clinical assessments

The WOMAC pain, stiffness and function subscales were included for the hip OA patients only. The patients evaluated their status on a 100 mm visual analogue scale, with 0 as anchor to no pain/stiffness/difficulty, and 100 as anchor to extreme pain/stiffness/difficulty [41]. For both patients and controls, hip ROM (°) in extension, flexion, abduction, adduction, internal rotation and external rotation was measured with a half-circle 1°-increment plastic goniometer. Finally, isokinetic muscle strength of the hip and knee joint extensors and flexors was evaluated from five repetitions at a velocity of 60° per second with a REV 9000 isokinetic dynamometer (Technogym, SpA, Gamboletta, Italy). The single highest achieved peak torque normalized to bodyweight (Nm/kilograms*100) was used as outcome measure. Detailed procedures for ROM and isokinetic muscle strength measurements have previously been described by Rydevik et al. [37].

#### Analysis

Analyses were done in SPSS 18.0 (SPSS Inc., Chicago, IL, US). All data were checked for normality with the Shapiro-Wilk test and Q-Q plots. To compare differences between patients and controls, and further between patients with MJS ≤/>2 mm, normally distributed variables were first assessed from the Levene’s test to decide whether p-values should be reported based on equality of variances with a Student’s t-test or non-equality with a Welch’s t-test. Non-normally distributed variables were evaluated with the independent Mann–Whitney U-test. Finally, binary logistic regression models with velocity as the predictor variable and key variables identified to be deviant between the hip OA patients and controls as dependent variables were calculated in order to control for velocity. Level of statistical significance was set to *p*<0.05.

## Results

Fourty-eight patients and 22 controls were included in the final analyses. The patients consisted of 19 men and 29 women, and the controls of 9 men and 13 women. The subject characteristics and secondary assessments describing level of symptoms and function for the overall group of hip OA patients and controls are presented in Table 1. No differences were found in anthropometric measures, but as expected the hip OA patients had significantly reduced hip ROM (*p*<0.001 to 0.001) in all directions except adduction. Reductions were most prominent in flexion (17.1°), internal rotation (15.3°), and external rotation (20.2°). Further, their knee extensor muscle strength was 33.5 Nm/BW lower compared to the controls (*p*=0.001).

**Table 1:** **Subject characteristics and hip function assessments**; **hip osteoarthritis patients and controls**

	**HIP OA PATIENTS(n=48)**		**CONTROLS(n=22)**		**MEAN DIFFERENCE(95% CI)**	**P-VALUE(TEST USED)**
	***Mean***	***SD***	***Mean***	***SD***		
**Age** (**years**)	59.1	9.48	58.5	8.79	0.6 (−4.21 to 5.33)	0.813^(□)^
**Height** (**cm**)	172.3	8.36	171.7	10.81	0.6 (−0.04 to 0.05)	0.811^(□)^
**Weight** (**kg**)	73.2	12.21	70.8	15.13	2.4 (−4.38 to 9.17)	0.483^(□)^
**Body Mass Index**	24.6	3.33	23.8	3.46	0.8 (−0.98 to 2.48)	0.388^(□)^
**Target joint MJS**^a^	1.9	1.05				
**Harris Hip Score**	78.2	8.15	99.5	1.19	−21.3 (−24.73 to −17.76)	<0.001^(□)^
**WOMAC**^b^						
WOMAC function	22.4	16.38				
WOMAC stiffness	32.1	22.92				
WOMAC pain	25.3	18.07				
**Hip range of motion** (°)						
Flexion	121.2	17.49	138.3	7.77	−17.1 (−25.16 to −8,86)	<0.001^(◊)^
Extension	0.8	8.56	9.9	5.15	−9.1(−13.00 to −4.78)	<0.001^(◊)^
Abduction	23.0	10.08	31.4	6.19	−8.4 (−12.60 to −2.90)	<0.001^(○)^
Adduction	23.5	7.48	25.4	5.39	−1.9 (−5.85 to 1.43)	0.317^(○)^
Internal rotation	31.4	15.01	46.7	11.74	−15.3 (−21.69 to −6.58)	0.001^(○)^
External rotation	24.3	12.91	44.5	7.49	−20.2 (−26.75 to −14.41)	<0.001^(◊)^
**Muscle strength** (**Nm**/BW*100^**c**^)						
Hip flexion	120.7	36.65	135.3	31.95	−14.6 (−33.40 to 4.20)	0.126^(□)^
Hip extension	197.2	68.96	223.4	57.35	−26.2 (−61.17 to 8.80)	0.140^(□)^
Knee flexion	87.7	27.96	98.3	25.18	−10.6 (−25.09 to 3.89)	0.149^(□)^
Knee extension	143.6	36.17	177.1	34.01	−33.5 (−52.47 to −14.56)	0.001^(□)^

^a^: Minimal joint space.

^b^: The Western Ontario and McMaster Universities Osteoarthritis Index.

^c^: Newton meter/body weight*100.

^□^: Student’s t-test.

^◊^: Welch’s t-test.

^○^: Mann–Whitney U-test.

**Figure 2:**
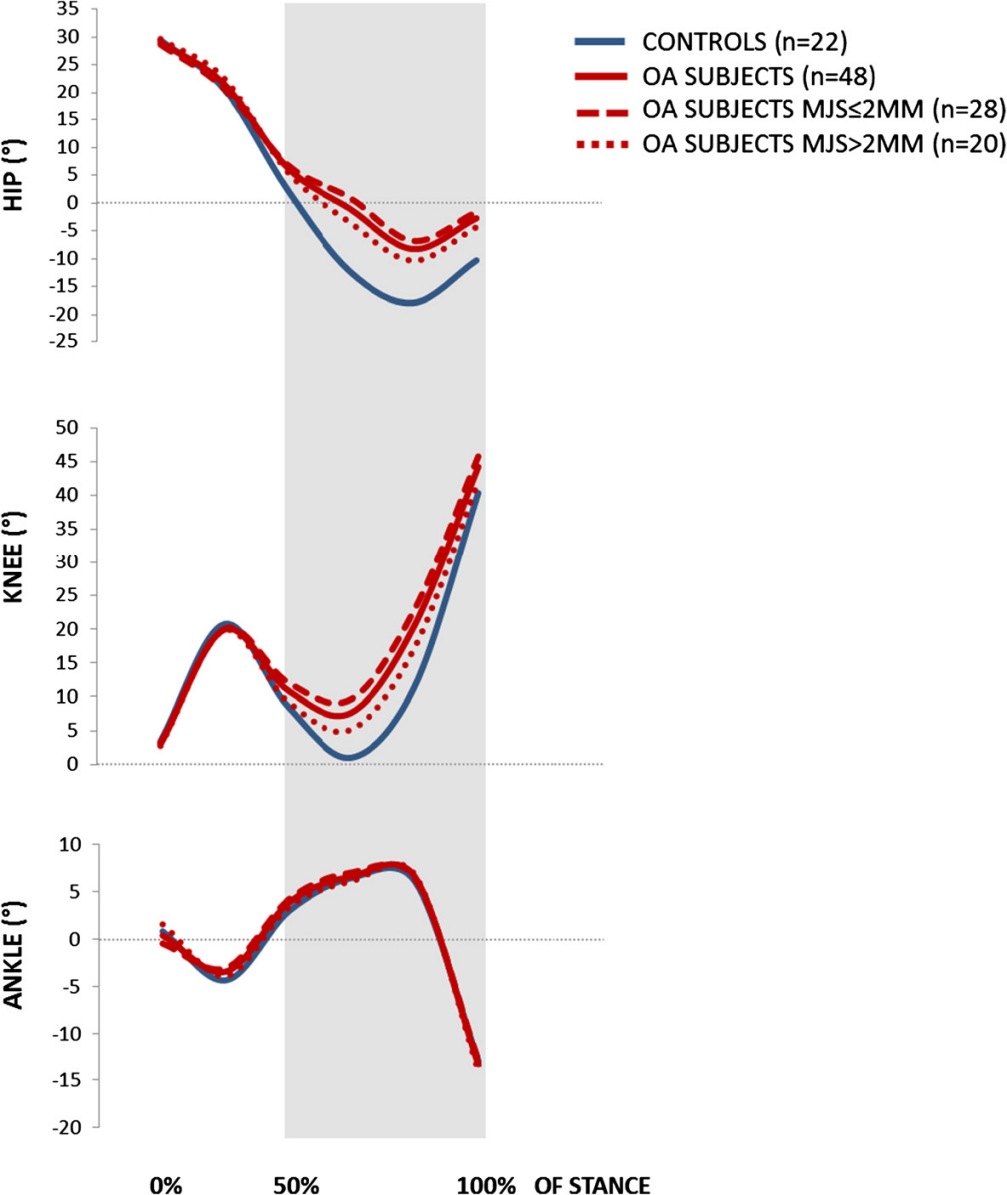
Joint excursion angles (°) of the hip, knee and ankle during stance phase of gait.

**Figure 3:**
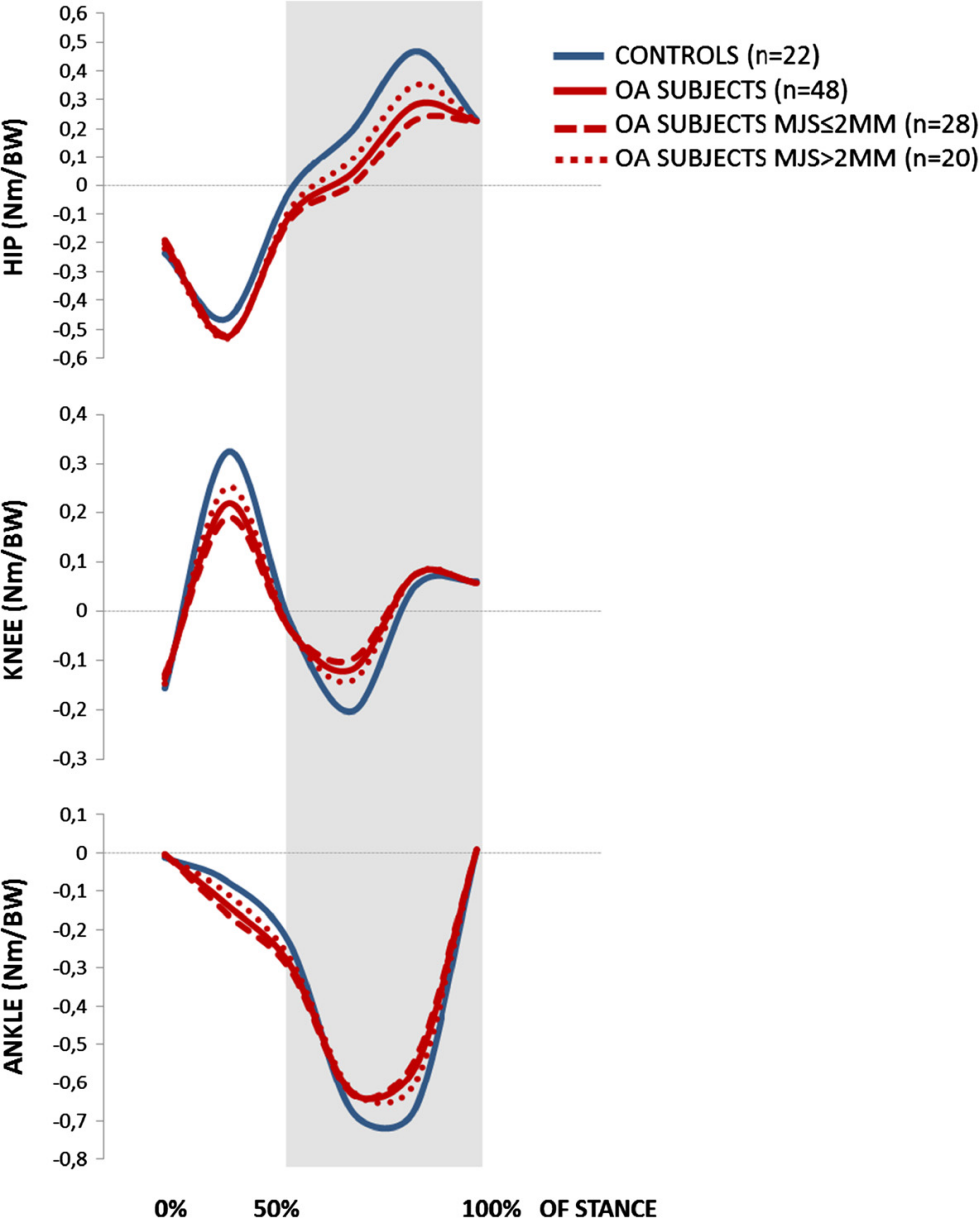
External joint moments (Nm/BW) for the hip, knee and ankle during stance phase of gait.

The hip, knee and ankle joint angles from initial contact to toe-off, and the corresponding joint moments, are shown in Figures 2 and 3, respectively. The figures include comparisons of both the overall group of patients versus controls, and between patients with MJS ≤/>2 mm.

Patients with hip OA walked significantly slower (*p*=0.002) than the controls, with a velocity of 1.53 (SD 0.2) compared to 1.65 (SD 0.2) meter per second. Statistical comparisons of joint excursion throughout stance, as well as specific angles and moments at the four defined events, are shown for the overall group of patients and controls in Table 2.

**Table 2:** Sagittal plane gait characteristics of all hip osteoarthritis patients and controls

	**HIP OA PATIENTS(n=48)**		**CONTROLS(n=22)**		**MEAN DIFFERENCE(95% CI)**	**P-VALUE(TEST USED)**
	***Mean***	***SD***	***Mean***	***SD***		
**GAIT VELOCITY** (**m**/**sec**)	1.53	0.146	1.65	0.150	−0.12 (−0.197 to −0.045)	0.002^(□)^
**KINEMATIC PARAMETERS** (°)						
**Hip excursion**	37.4	7.78	47.4	6.22	−10.0 (−13.79 to −6.25)	<0.001^(□)^
**Knee excursion**	41.2	6.08	36.9	7.07	4.3 (1.02 to 7.60)	0.011^(□)^
**Ankle excursion**	19.9	3.83	19.0	7.62	0.9 (−1.84 to 3.61)	0.519^(□)^
**Initial contact**						
Hip joint angle	29.1	5.15	29.5	7.43	−0.4 (−3.45 to 2.67)	0.825^(◊)^
Knee joint angle	3.0	3.86	3.5	3.91	−0.5 (−2.43 to 1.56)	0.669^(□)^
Ankle joint angle	0.4	3.19	0.9	3.69	−0.5 (−2.18 to 1.28)	0.604^(□)^
**Midstance**						
Hip joint angle	6.4	5.29	2.4	6.78	4.0 (0.97 to 6.92)	0.021^(◊)^
Knee joint angle	10.9	4.31	8.5	4.33	2.4 (0.17 to 4.60)	0.035^(□)^
Ankle joint angle	3.9	2.85	3.1	2.95	0.9 (−0.60 to 2.36)	0.238^(○)^
**Peak hip extension**						
Hip joint angle	−8.3	7.61	−17.9	6.85	9.6 (5.83 to 13.42)	<0.001^(□)^
Knee joint angle	20.5	8.22	11.5	6.04	9.0 (5.17 to 13.00)	<0.001^(□)^
Ankle joint angle	6.6	4.34	6.1	4.15	0.5 (−1.71 to 2.69)	0.658^(□)^
**Toe**-**off**						
Hip joint angle	−2.7	6.45	−10.3	7.00	7.6 (4.17 to 10.98)	<0.001^(□)^
Knee joint angle	44.2	5.26	40.3	6.21	3.9 (1.10 to 6.74)	0.009^(□)^
Ankle joint angle	−13.3	4.48	−12.9	7.38	−0.4 (−3.24 to 2.45)	0.783^(○)^
**KINETIC PARAMETERS** (**Nm**/**BW**^a^)						
**Initial contact**						
Hip joint moment	−0.202	0.0709	−0.234	0.0969	0.032 (−0.0095 to 0.0726)	0.130^(○)^
Knee joint moment	−0.134	0.0387	−0.156	0.0396	0.019 (−0.0001 to 0.0399)	0.051^(○)^
Ankle joint moment	−0.005	0.0127	−0.013	0.0089	0.008 (0.0017 to 0.1377)	0.002^(○)^
**Midstance**						
Hip joint moment	−0.105	0.0858	−0.023	0.7891	−0.082 (−0.1247 to −0.0386)	<0.001^(□)^
Knee joint moment	−0.037	0.0822	−0.020	0.0760	−0.017 (−0.5834 to 0.0244)	0.440^(□)^
Ankle joint moment	- 0.291	0.1035	−0.237	0.0761	−0.054 (−0.1037 to −0.0051)	0.007^(□)^
**Peak hip extension**						
Hip joint moment	0.284	0.1538	0.459	0.2017	−0.176 (−0.2631 to −0.0885)	<0.001^(□)^
Knee joint moment	0.075	0.0737	0.053	0.1136	0.022 (−0.0230 to 0.0674)	0.408^(◊)^
Ankle joint moment	−0.566	0.1584	−0.671	0.1976	0.105 (0.0173 to 0.1935)	0.035^(□)^
**Toe**-**off**						
Hip joint moment	0.226	0.0576	0.235	0.0804	−0.005 (−0.3902 to 0.02830)	0.780^(□)^
Knee joint moment	0.058	0.0196	0.061	0.0279	−0.003 (−0.1486 to 0.0083)	0.621^(◊)^
Ankle joint moment	0.008	0.0082	0.009	0.0086	−0.001 (−0.0045 to 0.0040)	0.892^(□)^

^a^: Newton meter/body weight.

^□^: Student’s t-test.

^◊^: Welch’s t-test.

^○^: Mann–Whitney U-test.

With regard to kinematics, hip and knee joint excursion was reduced by 10.0° (*p*<0.001) and 4.3° degrees (*p*=0.011), respectively. When adjusted for velocity, hip excursion was still significantly reduced (*p*=0.008), whereas knee excursion was not (*p*=0.161). Analyses at the specified events revealed that the observed differences in excursion stem from the latter 50% of the stance phase, where hip and knee joint extension was significantly reduced with 7.6° to 9.6° for the hip, and 3.9° to 9.0° for the knee (*p*-values from <0.001 to 0.009). The reduced kinematic hip extension was reflected in the kinetics, in that hip OA patients had a significantly reduced hip flexion moment at mid-stance with a mean difference of −0.082 Nm/BW, and at peak hip extension with a mean difference of −0.176 Nm/BW (both *p*<0.001). When controlled for velocity, these differences were still evident (*p*=0.015 and *p*=0.004, respectively). There were no differences in knee moments, but the plantarflexion ankle joint moment was significantly different with 0.008 Nm/BW, -0.054 Nm/BW and 0.105 Nm/BW at initial contact, midstance and peak hip extension, respectively (*p*-values 0.002 to 0.035).

Subject characteristics for patients with MJS ≤/>2 mm are given in Table 3. No statistical significant differences were established from the anthropometric measures, hip ROM, muscle strength or WOMAC; except for hip ROM abduction which was reduced by 4.8° (*p*=0.026) and adduction which was reduced by 3.8° (*p*=0.049) in the group with more severe ROA.

**Table 3:** **Subject characteristics and hip function assessments**; **hip osteoarthritis patients with MJS** ≤/>**2 mm**

	**MJS**^**a**^**≤2**.**0 mm(n=28)**		**MJS**^**a**^**>2**.**0 mm(n=20)**		**MEAN DIFFERENCE(95% CI)**	**P-VALUE(TEST USED)**
	***Mean***	***SD***	***Mean***	***SD***		
**Age** (**years**)	60.0	9.56	57.8	9.46	2.2 (−3.42 to 7.80)	0.437^(□)^
**Height** (**cm**)	173.5	9.36	170.5	6.57	3.0 (−0.02 to 0.08)	0.224^(◊)^
**Weight** (**kg**)	75.3	12.12	70.3	12.03	5.0 (−2.12 to 12.12)	0.164^(□)^
**Body Mass Index**	24.9	3.23	24.1	3.48	0.8 (−1.14 to 2.79)	0.404^(□)^
**Target joint MJS**^a^	1.2	0.71	2.9	0.43	−1.7 (−2.08 to −1.37)	<0.001^(◊)^
**Harris Hip Score**	77.3	8.26	79.5	8.02	−2.2 (−6.98 to 2.65)	0.370^(□)^
**WOMAC**^b^						
WOMAC pain	27.7	18.69	22.1	17.07	5.6 (−5.01 to 16.26)	0.272^(○)^
WOMAC function	25.9	17.96	17.5	12.76	8.4 (−1.05 to 17.83)	0.152^(○)^
WOMAC stiffness	34.5	23.0	28.7	22.96	5.8 (−7.78 to 19.31)	0.305^(○)^
**HIP ROM** (°)						
Flexion	117.7	17.46	126.7	16.58	−9.0 (−19.01 to 1.14)	0.081^(□)^
Extension	−0.3	7.95	2.5	9.32	−2.8 (−7.84 to 2.23)	0.267^(□)^
Abduction	21.5	10.68	26.3	8.69	−4.8 (−10.55 to −1.13)	0.026^(○)^
Adduction	21.9	7.02	25.7	7.49	−3.8 (−8.06 to −0.45)	0.049^(○)^
Internal rotation	28.9	14.87	35.9	14.80	−7.0 (−15.84 to 1.65)	0.094^(○)^
External rotation	22.0	11.65	27.5	14.17	−5.5 (−12.97 to 2.07)	0.151 ^□)^
**Muscle strength** (**Nm**/BW*100^c^)						
Hip flexion	118.9	43.66	123.2	24.57	−4.3 (−26.07 to 17.53)	0.669^(○)^
Hip extension	195.1	82.11	200.2	46.55	−5.1 (−46.20 to 35.89)	0.784^(○)^
Knee flexion	90.2	31.69	84.3	22.30	5.9 (−10.84 to 22.57)	0.483^(○)^
Knee extension	141.1	43.05	146.9	24.71	−5.8 (−27.47 to 15.85)	0.806^(□)^

^a^: Minimal joint space.

^b^: The Western Ontario and McMaster Universities Osteoarthritis Index.

^c^: Newton meter/body weight*100.

^□^: Student’s t-test.

^◊^: Welch’s t-test.

^○^: Mann–Whitney U-test.

Statistical comparisons of joint excursion throughout stance, as well as specific angles and moments at the four defined events for patients with MJS ≤/>2 mm are shown in Table 4.

**Table 4:** **Sagittal plane gait characteristics of hip osteoarthritis patients with MJS** ≤/>**2**.**0 mm**

	**MJS**^**a**^**≤2**.**0 mm(n=28)**		**MJS**^**a**^**>2**.**0 mm(n=20)**		**MEAN DIFFERENCE(95% CI)**	**P-VALUE(TEST USED)**
	***Mean***	***SD***	***Mean***	***SD***		
**VELOCITY** (**m**/**sec**)	1.56	0.122	1.50	0.159	−0.05 (−0.138 to 0.033)	0.223^(□)^
**KINEMATIC PARAMETERS** (°)						
**Hip excursion**	35.5	8.03	40.0	6.76	−4.5 (−8.95 to −0.08)	0.046^(□)^
**Knee excursion**	42.5	6.26	39.3	5.40	3.3 (−0.24 to 6.74)	0.067^(□)^
**Ankle excursion**	19.3	3.48	20.7	4.20	−1.5 (−3.72 to 0.76)	0.190^(□)^
**Initial contact**						
Hip joint angle	28.7	5.53	29.7	4.65	−1.0 (−4.04 to 2.07)	0.520^(□)^
Knee joint angle	3.2	4.17	2.8	3.48	0.4 (−1.87 to 2.73)	0.709^(□)^
Ankle joint angle	−0.4	3.09	1.6	3.00	−2.0 (−3.83 to −0.23)	0.028^(□)^
**Midstance**						
Hip joint angle	6.9	5.24	5.7	5.41	1.2 (−1.89 to 4.37)	0.431^(□)^
Knee joint angle	12.1	3.85	9.2	4.46	2.9 (0.46 to 5.30)	0.021^(□)^
Ankle joint angle	4.3	2.58	3.21	3.2	0.8 (−0.87 to 2.50)	0.336^(□)^
**Peak hip extension**						
Hip joint angle	−6.8	7.41	−10.3	7.60	3.5 (−0.88 to 7.94)	0.114^(□)^
Knee joint angle	22.9	8.34	17.2	6.94	5.7 (1.09 to 10.28)	0.016^(□)^
Ankle joint angle	6.4	4.11	6.8	4.76	−0.4 (−2.94 to 2.23)	0.782^(□)^
**Toe**-**off**						
Hip joint angle	−1.6	6.26	−4.3	6.55	2.7 (−1.04 to 6.5)	0.152^(□)^
Knee joint angle	45.7	4.81	42.1	5.22	3.6 (0.74 to 6.62)	0.015^(□)^
Ankle joint angle	−12.8	4.97	−13.9	3.69	1.1 (−1.53 to 3.77)	0.398^(□)^
**KINETIC PARAMETERS** (**Nm**/**BW**^b^)						
**Initial contact**						
Hip joint moment	−0.189	0.0609	−0.220	0.0813	0.031 (−0.0111 to 0.0714)	0.149^(□)^
Knee joint moment	−0.127	0.0338	−0.147	0.0431	0.020 (−0.0036 to 0.0411)	0.098^(□)^
Ankle joint moment	−0.004	0.0143	−0.007	0.0102	0.003 (−0.0038 to 0.0112)	0.092^(○)^
**Midstance**						
Hip joint moment	−0.119	0.0874	−0.083	0.0811	−0.036 (−0.8631 to 0.1371)	0.431^(□)^
Knee joint moment	−0.037	0.0769	−0.037	0.0912	0.000 (−0.4901 to 0.4896)	0.452^(○)^
Ankle joint moment	−0.304	0.1142	−0.274	0.0863	−0.029 (−0.0908 to 0.0311)	0.315^(○)^
**Peak hip extension**						
Hip joint moment	0.235	0.1419	0.352	0.1465	−0.117 (−0.2022 to −0.0327)	0.001^(○)^
Knee joint moment	0.076	0.0771	0.075	0.0707	0.001 (−0.0428 to 0.4497)	0.961^(□)^
Ankle joint moment	−0.539	0.1450	−0.601	0.1667	0.062 (−0.0307 to 0.1544)	0.186^(□)^
**Toe**-**off**						
Hip joint moment	0.225	0.0579	0.227	0.0587	−0.002 (−0.0364 to 0.0322)	0.903^(□)^
Knee joint moment	0.058	0.0174	0.057	0.0227	0.001 (−0.0103 to 0.1296)	0.822^(□)^
Ankle joint moment	0.009	0.0087	0.007	0.0073	0.002 (−0.0029 to 0.0067)	0.432^(□)^

^a^: Minimal joint space.

^b^:Newton meter/body weight.

^□^: Student’s t-test.

^◊^: Welch’s t-test.

^○^: Mann–Whitney U-test.

No difference (*p*=0.223) was found in gait velocity. Patients with MJS ≤2 mm revealed a 4.5° reduced hip excursion (*p*=0.046) compared to patients with MJS>2 mm. When adjusted for velocity, the difference was no longer significant (*p*=0.130). No significant kinematic differences were established for the hip joint at the specific events. Extension was significantly reduced only for the knee joint at midstance, with 2.9° (*p*=0.021), and at peak hip extension with 5.7° (*p*=0.016). The hip flexion moment at peak hip extension was, however, significantly lower in patients with MJS ≤2 mm, with a mean difference of −0.117 Nm/BW (*p*<0.001). This difference was persistent after controlling for velocity (*p*=0.028). No differences were established for knee- or ankle moments.

## Discussion

The main findings of this study were that compared to controls, patients with hip OA with mild to moderate symptoms walked with significantly reduced velocity, and revealed significantly reduced hip and knee joint excursion, specifically manifested as reduced extension in the hip and knee joint during the latter 50% of the stance phase. Thus, our hypothesis that hip OA patients in this early stage of disease would reveal reduced hip excursion during stance and a reduced gait velocity compared to healthy subjects was confirmed. Our secondary hypothesis, that hip OA patients with severe ROA defined from a MJS ≤2 mm would reveal reduced hip excursion during stance and reduced gait velocity compared to patients with MJS >2 mm, was only partly confirmed; as these patients had significantly reduced hip excursion during stance, but no difference in gait velocity.

The rationale for this study was the lack of consensus on the identification of specific gait parameters in patients with hip OA, especially with regard to patients in the early stage of disease. The included patients in this study had a mean (SD) HHS score of 78.2 (8.2), defining them as non-eligible for THR in our institution, and a mean (SD) WOMAC pain subscore of 25.3 (18.1), reflecting mild to moderate pain [42]. In our clinic, the orthopedic surgeons use ≤2 mm MJS as their clinically anchored cut-off when categorizing patients with hip OA from their level of radiographic progression. We therefore defined this as the cut-off value for subcategorizing our cohort. There is no absolute consensus on categorizing radiographic severity based on MJS in hip OA. However, several studies state ≤2.5 mm MJS as a minimum for defined ROA, and a MJS ≤1.5 mm as severe ROA [1,2,43-45]. Reijman and colleagues [46] more explicitly suggest MJS ≤2.5 mm as intermediate, ≤2 mm as moderate and ≤1.5 mm as severe ROA, respectively. The hip OA patients included in this study were, thus, overall ratified to having a mild to moderate level of disease, assessed both from symptoms and radiographs; and the subjects classified to have more severe ROA as having at least moderate radiographic changes.

The identification of reduced velocity and reduced hip and knee joint extension during the latter 50% of the stance phase as key gait features in our cohort, confirmed that the biomechanical deviations most commonly described in severe hip OA gait are manifest already at an early stage of disease. To our knowledge, no more than two other studies [27,28] have reported 3D gait analysis data of patients at a comparable stage of hip OA, and also compared them to controls. Of these, only the study by Dujardin and colleagues [27] had a sample size comparable to ours. Unfortunately, their study was published in French, so we were unable to weigh our findings against theirs. In line with our findings, the other study by Watelain et al. [28] identified reduced velocity and reduced hip excursion as characteristics of gait in patients with early stage hip OA. However, their sample size was considerably smaller than ours. Thus, our study adds clinically relevant knowledge on biomechanics during gait in a population of hip OA patients that previously has been given little attention.

Our hip OA patients walked with significantly reduced velocity compared to the controls. Still, their walking speed was faster than what have commonly been reported, compared both to age-related normative data [47,48] and studies including patients with severe hip OA [12,49]. A plausible cause for the latter is the fact that we included patients with mild to moderate symptoms, in contrast to most studies that have focused on patients in the end-stage of disease. Higher walking velocity has been shown to increase joint moments [50] and accentuate pathological changes in patients with severe hip OA [51]. Thus, it can be questioned whether observed biomechanical changes in hip OA patients can be explained merely from their reduced gait speed. However, reduced velocity appears inherently linked to disease progression, and can as such be regarded to be an intrinsic key feature of hip OA gait. Thus, it is not straightforward to consider velocity solely as a “disturbing” variable, that should be controlled for in a regular analysis of variance, leaving the unadjusted analyses out [26]. From a clinical perspective, controlling for velocity can give diminutive meaning; as it is inapplicable to just tell the patients to walk faster in order to reduce their joint moments. For these reasons, we performed the main analyses unadjusted for velocity; but included a supplementary binary logistic regression model to reveal whether the observed key differences in hip and knee excursion and hip joint moments were persistent after adjusting for velocity. These analyses confirmed that the reductions in hip excursion and hip joint moments in hip OA patients compared to controls were persistent regardless of velocity; whereas knee excursion was not. The unadjusted and adjusted analyses together thus strengthened the identification of reduced hip extension during excursion and hip flexion moments as key features of gait in hip OA patients with mild to moderate symptoms.

Abnormal joint loading has been shown to contribute to detrimental shear stresses as well as disruption and loss of cartilage, and is considered to be an important mechanism of lower limb OA pathogenesis [52-59]. Accumulated inadequate loads may further play a role in disease progression, as they can facilitate enlargement of the joint surface that is worn down [29,59,60]. A relevant factor in this aspect is the reduced range of passive hip joint extension we found for the hip OA patients. Static contractures that increase the stiffness of the hip joint have long been suggested to limit hip extension during the late phase of stance [23]. The hip OA patients in our study had a mean of 0.8° (8.7) passive hip ROM extension; a mean reduction of 9.1° compared to the controls (*p*<0.001). This was reflected during gait, where the hip OA patients had 9.6° less peak hip extension than the controls. Simonsen et al. [61] recently reported that walking with the upper body inclined was associated with both a significant increase in hip joint flexion angle and a significantly reduced hip flexion moment in the latter phase of stance. As our experimental set-up did not include an upper body model, we could not report the positioning of the upper body in our subjects. However, we found the exact same pattern of both reduced hip extension angle and reduced hip flexion moment during late stance in our cohort. The verification of these biomechanical deviations already in hip OA patients with mild to moderate symptoms, may reflect an unfavorable stress distribution that possibly can contribute to facilitated disease progression.

In correspondence with the hip joint, reduced extension during the latter 50% of the stance phase was found also in the adjacent knee when we compared the hip OA patients to the controls (*p*-values between <0.001 and 0.035). Further, a significant reduction in isokinetic knee extension strength was established between the overall group of patients and the controls (*p*=0.001). It is clinically important to be aware that such deviations will influence knee joint loading, and over time may contribute to the development of secondary degenerative changes also in the knee joint. As the patients in this study were excluded if they had any form of knee pain or dysfunction, including restrictions in passive knee ROM, the observed differences at the knee joint are likely to be adaptive consequences of the structural changes and pain in the hip joint. This was further supported by the finding that in contrast to the hip joint excursion, the knee joint excursion difference was no longer significant when adjusting for velocity.

To what extent the level of ROA is reflected in biomechanics during gait in hip OA patients non-eligible for THR, has never been investigated. When subcategorizing the hip OA patients from their level of ROA, the patients with ≤2 mm MJS were classified as having severe ROA (mean MJS of 1.2mm), and the patients with >2 mm MJS as having intermediate ROA (mean MJS of 2.9 mm) [46]. The hip and knee kinematic joint excursion curves illustrated that patients with MJS ≤2 mm revealed the largest deviations. However, this was not reflected in the functional or clinical assessments. No differences were evident between patients with MJS ≤/>2 mm in either of the WOMAC subscales. It has been stated that ROM restrictions are larger in patients with more severe ROA [62], but we did not find statistical significant differences in hip ROM flexion or extension between the two subgroups. However, the lack of significance must be interpreted with caution, due to the relatively low number of subjects in each group. It cannot be ruled out that the 9° difference in hip flexion and the 7° difference in internal rotation may be of clinical importance. Finally, we found no differences in isokinetic hip muscle strength. However, patients with MJS ≤2 mm revealed a highly significant reduced hip flexion moment at peak hip extension (*p*=0.001), that still was significantly reduced when controlling for velocity (*p*=0.028). This may indicate non-optimal use of the hip extensor muscles that was manifested during walking; but not during the isokinetic test. In sum, our findings suggested that more severe ROA resulted in more distinct gait alterations in the sagittal plane, even though there were no significant differences in symptoms or function. This emphasizes the importance of supplementing self-reported and clinical outcome measures with assessments that quantify function and gait [63], when trying to understand how altered gait can influence disease progression – or vice versa.

Overall, the findings of this study are in line with previous studies suggesting reduced velocity, reduced sagittal plane joint excursion and a reduced hip flexion moment in the late stance phase as key features of hip OA gait. What is new from our study, is that these hallmarks are evident already in hip OA patients at an early stage of symptoms. These patients are not eligible for total hip replacement. Rather; they should be considered as primary candidates for exercise therapy. In this context, the identification of early stage gait alterations is clinically important, as it may contribute to enable development of targeted treatment interventions. To what extent the observed alterations were primarily caused by – or a result from – the level of pain, changes in bone and cartilage morphology, muscle weakness or reduced passive ROM, is still unknown. Thus, it remains a question whether the gait characteristics described in this study may drive further progression of either symptomatic or radiographic hip OA; or primarily reflect adaptations to the constraints posed by the degenerative changes in the joint. Hence, future studies should investigate with particular interest the relationship between these key biomechanical features and different clinical and functional outcomes.

There are some limitations to this study that need to be addressed. First, only sagittal plane gait characteristics were reported, as we wanted to emphasize hip excursion, and in particular hip extension, in this plane. A comprehensive gait analysis should however include also frontal and transversal plane kinematics and kinetics. Further, the included analyses between the two pre-defined subgroups of patients with MJS ≤/>2 mm were not statistically corrected for multiplicity. In addition, the subgroup sample sizes were smaller. Our findings related to the secondary aim of this study should therefore be regarded as an explorative supplement to the main analysis, and as such be interpreted as hypothesis-generating rather than conclusive [64].

## Conclusions

Patients with hip OA with mild to moderate symptoms walked with reduced velocity compared to healthy controls, and revealed evident alterations in the latter 50% of the stance phase of gait. Reduced extension during hip and knee excursion and a reduced hip flexion moment were the main characteristics. From supplementary analyses of the two pre-defined subgroups of patients with different levels of ROA, this study generated the hypothesis that the observed characteristics were more pronounced in hip OA patients with ≤2 mm MJS.

## Abbreviations

HHS: Harris Hip Score; MJS: Minimal joint space; Nm/BW: Newton-meter normalized to bodyweight; OA: Osteoarthritis; ROA: Radiographic osteoarthritis; ROM: Range of motion; SD: Standard deviation; THR: Total hip replacement; WOMAC: Western Ontario and McMaster Universities Osteoarthritis Index.

## Competing interests

The authors declare that they have no competing interests.

## Authors’ contributions

All authors contributed to the idea and the design of the study, contributed to drafting of the article including discussions regarding the analyses and results, and read and approved the final manuscript. LF had the main responsibility for the data collection. IE performed all statistical analyses and had the main responsibility for writing the manuscript.

## Pre-publication history

The pre-publication history for this paper can be accessed here:

http://www.biomedcentral.com/1471-2474/13/258/prepub
