# Erratum: Sagittal plane gait characteristics in hip osteoarthritis patients with mild to moderate symptoms compared to healthy controls: a cross-sectional study

**DOI:** 10.1186/s12891-015-0483-8

**Published:** 2015-03-13

**Authors:** I Eitzen, L Fernandes, L Nordsletten, M A Risberg

**Affiliations:** The Norwegian Research Center for Active Rehabilitation (NAR)/Orthopaedic Department, Oslo University Hospital, Oslo, Norway; Orthopaedic Department, Oslo University Hospital, Oslo, Norway; The Norwegian Research Center for Active Rehabilitation/The Norwegian School of Sports Sciences/Orthopaedic Department, Oslo University Hospital, Oslo, Norway

During the review process of a subsequent paper, we have unfortunately discovered that our original interpretation of the sagittal plane hip joint moment in our previously published original article [1] was incorrect. In the original publication, we reported that individuals with mild to moderate hip osteoarthritis revealed a reduced sagittal plane external hip flexion moment. However, the sagittal plane external hip joint moment shifts from flexion to extension during stance. When the hip joint is extended above neutral position (0 degrees), the external moment should no longer be interpreted as a flexion moment, but as an extension moment. As the mean hip joint angle in our material do exceed 0 degrees at peak hip extension and toe-off; the force passes posterior to the joint center. Thus, our findings should have been reported as an external hip extension moment, not an external hip flexion moment.

Our misinterpretation in the original article has affected our results in that it should have been reported in the Results and Conclusion that the hip OA patients revealed a reduced external hip extension moment at peak hip extension, not a reduced external hip flexion moment. Consequently, the associated graph in Figure 3, and corresponding numbers in Table 2 and 4, should have been presented in the opposite direction with regard to negative and positive values. Corrected versions of Figure 3 and Tables 2 and 4 are provided in this erratum.

**Figure 3:**
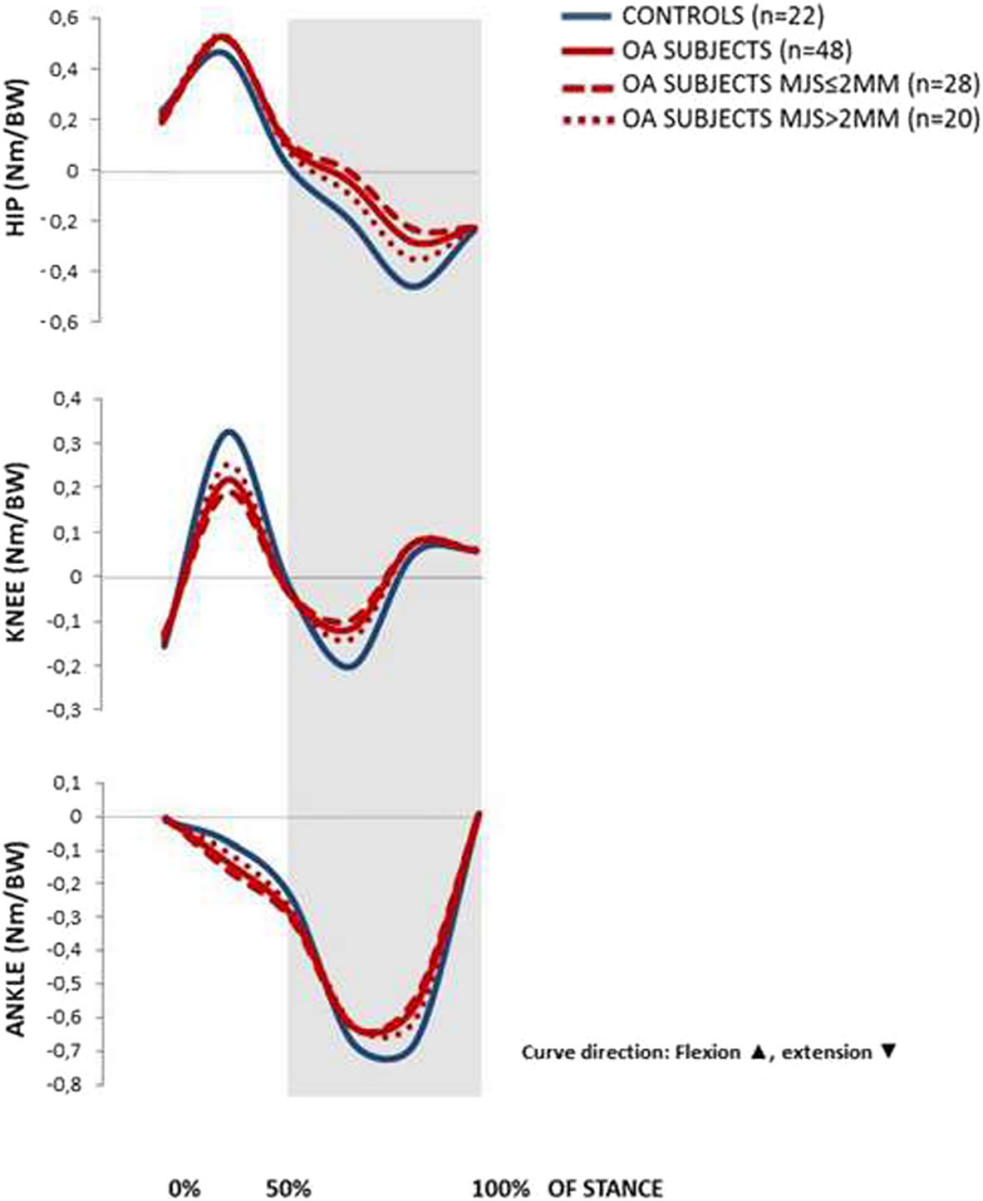
.

**Table 2:** **Sagittal plane gait characteristics of all hip osteoarthritis patients and controls correctedTable**

	**Hip OA patients** ** (n = ** **48)**		**Controls** ** (n = ** **22)**		**Mean difference** ** (95% ** **CI)**	**P**-**value** ^**(TEST USED)**^
	***Mean***	***SD***	***Mean***	***SD***		
**GAIT VELOCITY** **(** **m** **/** **sec)**	1.53	(0.146)	1.65	(0.150)	−0.12 (−0.197 to −0.045)	0.002^(□)^
**KINEMATIC PARAMETERS (°)**						
**Hip excursion**	37.4	7.78	47.4	6.22	−10.0 (−13.79 to −6.25)	<0.001^(□)^
**Knee excursion**	41.2	6.08	36.9	7.07	4.3 (1.02 to 7.60)	0.011^(□)^
**Ankle excursion**	19.9	3.83	19.0	7.62	0.9 (−1.84 to 3.61)	0.519^(□)^
**Initial contact**						
Hip joint angle	29.1	5.15	29.5	7.43	−0.4 (−3.45 to 2.67)	0.825^(◊)^
Knee joint angle	3.0	3.86	3.5	3.91	−0.5 (−2.43 to 1.56)	0.669^(□)^
Ankle joint angle	0.4	3.19	0.9	3.69	−0.5 (−2.18 to 1.28)	0.604^(□)^
**Midstance**						
Hip joint angle	6.4	5.29	2.4	6.78	4.0 (0.97 to 6.92)	0.021^(◊)^
Knee joint angle	10.9	4.31	8.5	4.33	2.4 (0.17 to 4.60)	0.035^(□)^
Ankle joint angle	3.9	2.85	3.1	2.95	0.9 (−0.60 to 2.36)	0.238^(○)^
**Peak hip extension**						
Hip joint angle	−8.3	7.61	−17.9	6.85	9.6 (5.83 to 13.42)	<0.001^(□)^
Knee joint angle	20.5	8.22	11.5	6.04	9.0 (5.17 to 13.00)	<0.001^(□)^
Ankle joint angle	6.6	4.34	6.1	4.15	0.5 (−1.71 to 2.69)	0.658^(□)^
**Toe**-**off**						
Hip joint angle	−2.7	6.45	−10.3	7.00	7.6 (4.17 to 10.98)	<0.001^(□)^
Knee joint angle	44.2	5.26	40.3	6.21	3.9 (1.10 to 6.74)	0.009^(□)^
Ankle joint angle	−13.3	4.48	−12.9	7.38	−0.4 (−3.24 to 2.45)	0.783^(○)^

^a^: Newton meter/body weight.

^□^: Student’s t-test.

^◊^: Welch’s t-test.

○: Mann-Whitney U-test.

**Table 4:** **Sagittal plane gait characteristics of hip osteoarthritis patients with MJS** ≤/>**2.0 mm corrected**

	**MJS** ^**a**^ **≤2.0 mm** **(n = ** **28)**		**MJS** ^**a**^ **>2.0 mm** **(n = ** **22)**		**MEAN DIFFERENCE** ** (95% ** **CI)**	**P**-**VALUE** ^**(TEST USED)**^
	***Mean***	***SD***	***Mean***	***SD***		
**VELOCITY** **(** **m**/**sec** **)**	1.56	0.122	1.50	0.159	−0.05 (−0.138 to 0.033)	0.223^(□)^
**KINEMATIC PARAMETERS (°)**						
**Hip excursion**	35.5	8.03	40.0	6.76	−4.5 (−8.95 to −0.08)	0.046^(□)^
**Knee excursion**	42.5	6.26	39.3	5.40	3.3 (−0.24 to 6.74)	0.067^(□)^
**Ankle excursion**	19.3	3.48	20.7	4.20	−1.5 (−3.72 to 0.76)	0.190^(□)^
**Initial contact**						
Hip joint angle	28.7	5.53	29.7	4.65	−1.0 (−4.04 to 2.07)	0.520^(□)^
Knee joint angle	3.2	4.17	2.8	3.48	0.4 (−1.87 to 2.73)	0.709^(□)^
Ankle joint angle	−0.4	3.09	1.6	3.00	−2.0 (−3.83 to −0.23)	0.028^(□)^
**Midstance**						
Hip joint angle	6.9	5.24	5.7	5.41	1.2 (−1.89 to 4.37)	0.431^(□)^
Knee joint angle	12.1	3.85	9.2	4.46	2.9 (0.46 to 5.30)	0.021^(□)^
Ankle joint angle	4.3	2.58	3.21	3.2	0.8 (−0.87 to 2.50)	0.336^(□)^
**Peak hip extension**						
Hip joint angle	−6.8	7.41	−10.3	7.60	3.5 (−0.88 to 7.94)	0.114^(□)^
Knee joint angle	22.9	8.34	17.2	6.94	5.7 (1.09 to 10.28)	0.016^(□)^
Ankle joint angle	6.4	4.11	6.8	4.76	−0.4 (−2.94 to 2.23)	0.782^(□)^
**Toe**-**off**						
Hip joint angle	−1.6	6.26	−4.3	6.55	2.7 (−1.04 to 6.5)	0.152^(□)^
Knee joint angle	45.7	4.81	42.1	5.22	3.6 (0.74 to 6.62)	0.015^(□)^
Ankle joint angle	−12.8	4.97	−13.9	3.69	1.1 (−1.53 to 3.77)	0.398^(□)^
**KINETIC PARAMETERS** **(** **Nm**/**BW** ^**b**^ **)**						
**Initial contact**						
Hip joint moment	0.189	0.0609	0.220	0.0813	−0.031 (0.0111 to −0.0714)	0.149^(□)^
Knee joint moment	−0.127	0.0338	−0.147	0.0431	0.020 (−0.0036 to 0.0411)	0.098^(□)^
Ankle joint moment	−0.004	0.0143	−0.007	0.0102	0.003 (−0.0038 to 0.0112)	0.092^(○)^
**Midstance**						
Hip joint moment	0.119	0.0874	0.083	0.0811	0.036 (0.8631 to −0.1371)	0.431^(□)^
Knee joint moment	−0.037	0.0769	−0.037	0.0912	0.000 (−0.4901 to 0.4896)	0.452^(○)^
Ankle joint moment	−0.304	0.1142	−0.274	0.0863	−0.029 (−0.0908 to 0.0311)	0.315^(○)^
**Peak hip extension**						
Hip joint moment	−0.235	0.1419	−0.352	0.1465	0.117 (0.2022 to 0.0327)	0.001^(○)^
Knee joint moment	0.076	0.0771	0.075	0.0707	0.001 (−0.0428 to 0.4497)	0.961^(□)^
Ankle joint moment	−0.539	0.1450	−0.601	0.1667	0.062 (−0.0307 to 0.1544)	0.186^(□)^
**Toe**-**off**						
Hip joint moment	−0.225	0.0579	−0.227	0.0587	0.002 (0.0364 to −0.0322)	0.903^(□)^
Knee joint moment	0.058	0.0174	0.057	0.0227	0.001 (−0.0103 to 0.1296)	0.822^(□)^
Ankle joint moment	0.009	0.0087	0.007	0.0073	0.002 (−0.0029 to 0.0067)	0.432^(□)^

^a^: Minimal joint space.

^b^:Newton meter/body weight.

^□^: Student’s t-test.

^◊^: Welch’s t-test.

^○^: Mann–Whitney U-test.

Our overall findings, that hip OA patients reveal altered gait kinematics (reduced hip and knee excursion) compared to controls, and that those with more severe radiographic OA reveal larger deviations than those with less severe radiographic OA, remain. All other original graphs and tables are correct. Thus, the mistakes in the interpretation of the hip joint moment do not affect the soundness of the original study.

We apologize for the inconvenience this error in our original interpretation of the data in the original analysis may have caused.
